# *Micropeplus liweiae* sp. n., a new species from Sichuan, China (Coleoptera, Staphylinidae, Micropeplinae)

**DOI:** 10.3897/zookeys.775.22620

**Published:** 2018-07-18

**Authors:** Cheng-Bin Wang, Ri-Xin Jiang, Jiang Zhu

**Affiliations:** 1 Innovation College, Mianyang Normal University, 166 Mianxing West Road, Mianyang 621000, Sichuan Province, PR China; 2 Department of Biology, Shanghai Normal University, 100 Guilin Road, Shanghai 200234, PR China; 3 College of Agriculture and Biology, Zhongkai University of Agriculture and Engineering, 388 Guangxin Road, Guangzhou 510550, Guangdong Province, PR China

**Keywords:** China, Micropeplinae, *Micropeplus*, new species, Staphylinidae, taxonomy

## Abstract

A new species of micropepline beetle is described from Sichuan, China, *Micropeplus liweiae* **sp. n.** (Coleoptera, Staphylinidae). Important morphological characters of the new species are illustrated by colour plates.

## Introduction

*Micropeplus* Latreille, 1809 is the most speciose genus of Micropeplinae (Coleoptera, Staphylinidae), with members distributed in Ethiopian, Nearctic, Neotropical, Oriental and Palaearctic Regions. Campbell (1968, 1992, 1995) established eight species-groups to classify the species of the genus.

In the fauna of China, 22 species had been recorded before this study (Herman 2001; Schülke and Smetana 2015; Grebennikov and Smetana 2015; Zheng et al. 2016). Grebennikov and Smetana (2015) revealed the amazing hyper diversity of Micropeplinae in Southwest China. In this paper, *Micropeplus liweiae* sp. n., a new species belonging to the *staphylinoides* species-group is described and illustrated from Sichuan Province, China. The type specimens were collected under a rock in an alpine meadow.

## Materials and methods

Specimens were relaxed and softened in a hot saturated solution of potassium hydroxide for 4 minutes (for dry mounted specimens) or 8 minutes (for alcohol-preserved specimens), and then transferred to distilled water to rinse the residual potassium hydroxide off and stop any further bleaching. The softened specimens were moved into glycerin and dissected there to observe morphological details. After examination, the body parts were mounted on a glass slip with Euparal Mounting Medium for future studies. Habitus photographs were taken using a Canon MP-E 65 mm f/2.8 1-5X macro lens on a Canon 7D camera, and a Canon MT-24EX macro twin light flash was used as light source. Observations, photographs, and measurements of morphological details were performed using an Olympus CX31 microscope with a Canon G9 camera. The final deep focus images were created with Zerene Stacker 1.04 stacking software. Adobe Photoshop CS6 was used for post processing.

The material examined for this study is deposited in the following collections (with names of curators in parentheses): **SNUC** – Insect Collection of Shanghai Normal University, Shanghai, China (L. Tang); **SYSB** – Museum of Biology, Sun Yat-sen University, Guangzhou, China (F.-L. Jia).

Measurement criteria in millimetres (mm) are used as follows:

**Body length** length between the anterior apex of clypeus and the abdominal apex along the midline.

**Elytral length** length between the basal border and the apex of elytra along suture.

**Elytral width** widest part of both elytra combined.

**Fore body** length between the anterior apex of clypeus and the apex of elytra along the midline.

**Head length** length between the anterior apex of clypeus and the posterior margin of occiput along the midline.

**Head width** widest part of head (including compound eyes).

**Pronotal length** length of the pronotum along the midline.

**Pronotal width** widest part of pronotum.

## Results

### Genus *Micropeplus* Latreille, 1809

Vernacular name: 铠甲属

#### Micropeplus liweiae sp. n.

Taxon classificationAnimaliaColeopteraStaphylinidae

http://zoobank.org/A6D7C159-AA58-4903-8E47-E85A0F2E5CA5

**Figure 1.**
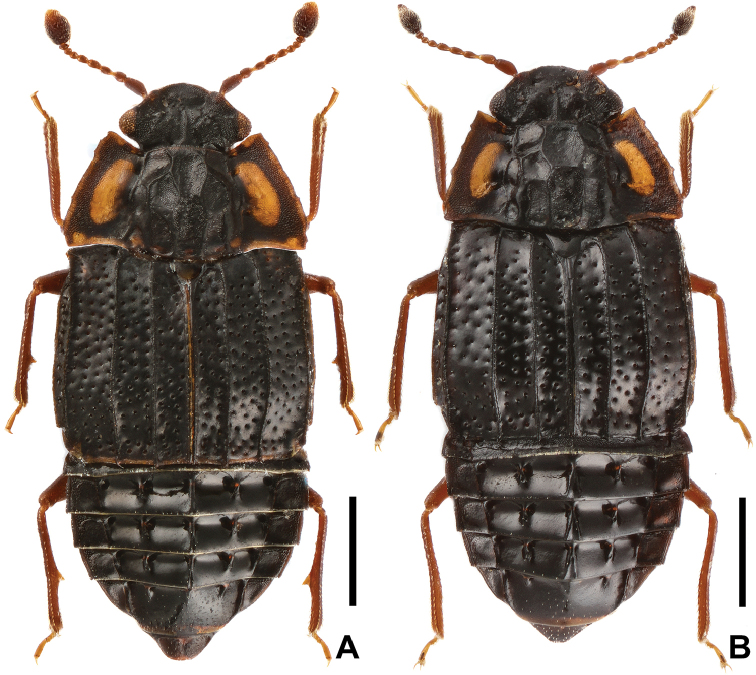
Habitus of *Micropeplus liweiae* sp. n. (dorsal view): **A** holotype ♂ **B** paratype ♀. Scale bars: 0.5 mm.

**Figure 2.**
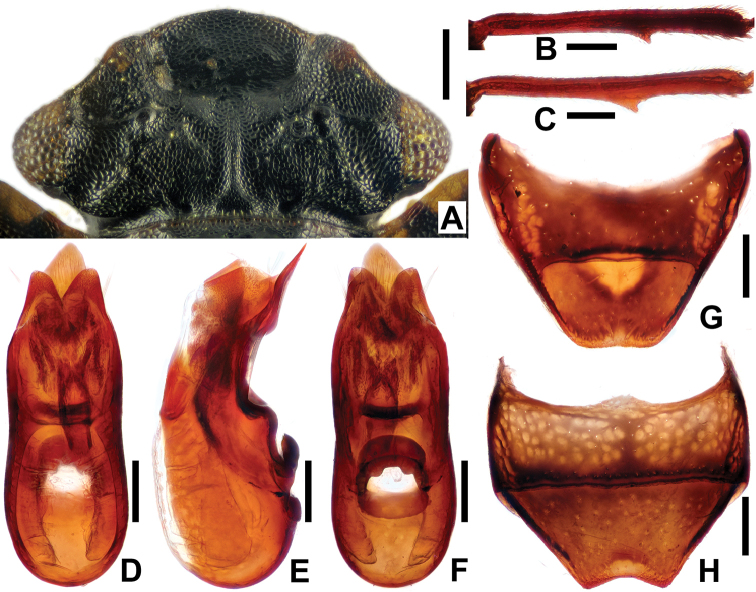
*Micropeplus liweiae* sp. n., holotype ♂: **A** head **B** mesotibia **C** metatibia **D–F** aedeagus **G** abdominal tergite VIII **H** abdominal ventrite VIII. Scale bars: 0.5 mm (**A**); 0.1 mm (**B–H**). (**A–D, G** dorsal view **E** lateral view **F, H** ventral view).

Figs 1A, B ; 2A–H Vernacular name: 力伟铠甲

##### Type material.

**Holotype**: ♂, CHINA, Sichuan: Xiaojin County [小金县], near Siguniang Shan [接近四姑娘山], 30.921623°N, 102.889709°E, under rock, 4258m, 14.VII.2017, Jiang Zhu leg. (SYSB). **Paratype**: 1♀, same data as holotype (SNUC).

##### Diagnosis.

This new species is very similar to *Micropeplus songi* Zheng, Li & Yan, 2014 from Mt. Wahui [瓦灰山], Sichuan, but it is easy to distinguish it from the latter by a combination of the following characteristics: head with microreticulate surface; elytral punctures moderate-sized, distinctly smaller but more numerous than that of *M. songi*; elytral interspace I with two to three rows, II with three to four rows, III with four rows, IV with one row and V with three rows; metathoracic wings fully developed; aedeagal parameres with two long setae at apex.

##### Description.

*Male*. Body small, 2.81 mm long. Length (mm) of different body parts: head (0.31), pronotum (0.55), elytra (1.05), fore body (1.91), aedeagus (0.58). Width (mm): head (0.64), pronotum (1.16), elytra (1.22). (Head width)/(pronotal width) = 0.55, (pronotal length)/(elytral length) = 0.52.

Habitus (Figure 1A) elliptical, generally convex and sublustrous. Head, disc of pronotum, elytra and abdomen blackish brown to black; basal eight antennomeres and apical half of ultimate antennomere, maxillary and labial palpi, legs, and sides of pronotum yellowish brown to brown.

Head (Figure 2A) transverse, widest across eyes, width/length = 2.06. Clypeus with anterior margin broadly subrounded. Vertex with a longitudinal carina along midline in basal half; area on both sides of carina weakly impressed; one fine transverse carina and two oblique carinae at middle of inner side of each eye; spaces between carinae microreticulate. Eyes distinctly prominent. Antennae have 9 antennomeres with single-segmented clubs; antennomere I more robust than other antennomeres except IX, and about equal to length of II+III combined; II narrowed apically, shorter and slightly narrower than I; III–VI longer than wide, narrower than II, III–V subequal and longer than VI; VII and VIII transverse; IX largest and oval, covered with dense pubescence.

Pronotum subtrapezoidal, widest just before hind angles, width/length = 2.11. Sides almost obliquely linear, gradually narrowing from posterior to anterior, with ca. 4 small teeth; anterior margin broadly and distinctly emarginate and slightly arched in middle; posterior margin bisinuate; anterior angles distinctly projected forwards; posterior angles nearly rectangular; surface finely granulate; lateral areas broadly explanate; median area elevated dorsally, with fourteen cells enclosed by costae (including two small ones after anterior margin).

Scutellum shield-like with finely granulate surface.

Elytra subquadrate, width/length = 1.16, widest at about apical 2/5. Each elytron with 6 costae, one sutural, two discal, one humeral, one pseudepipleural (straight, distinctly separated anteriorly and posteriorly from epipleural costa) and one epipleural; interspaces between costae with irregular rows of moderate-sized punctures, interspace I with two to three rows, II with three to four rows, III with four rows, IV with one row and V with three rows. Metathoracic wings fully developed.

Metastemum with elongate median impression, wide and deep at base, weakening anteriorly, about half as long as metasternum. Abdomen gradually narrowed towards segment VI and abruptly narrowed from VII to apical end; tergites III–VII each strongly and transversely depressed in basal half, III with one short median longitudinal carina, IV to VI each with three longitudinal carinae almost throughout length of tergite, VII with three abbreviated carinae in about basal 1/3, VIII (Figure 2G) with apical edge almost straight; sternite VIII (Figure 2H) with apical edge distinctly and subroundly emarginated, and semilunarly depressed before emargination.

Protibia without tooth on medial margin; meso- (Figure 2B) and metatibiae (Figure 2C) each armed with a large subtriangular tooth at about apical 1/3 of medial margins.

Aedeagus (Figure 2D–F) stout; median lobe weakly bent ventrad in apical part; parameres fused with median lobe, each with two long setae at apex.

*Female*. Similar to male in general appearance (Figure 1B), including anterior margin of clypeus which lacking sexual dimorphism, but distinct in the following characters: meso- and metatibiae without tooth on medial margins, and sternite VIII without emargination at apical edge.

##### Habitat.

**Figure 3.**
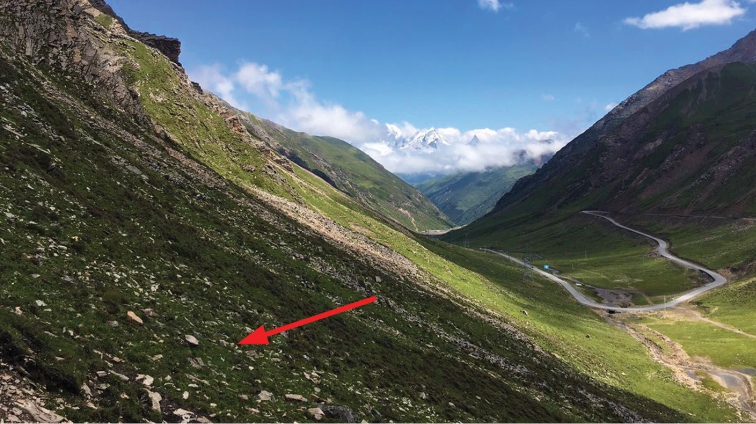
Habitat of *Micropeplus liweiae* sp. n. The new species was collected under a rock (red arrow) in an alpine meadow of Xiaojin, Sichuan.

Specimens were found under a rock in an alpine meadow (Figure 3), with high altitude over 4000 m.

##### Distribution.

China (Sichuan).

##### Etymology.

The specific epithet is dedicated to Ms. Li-Wei Liu, the mother of Jiang-Zhu (the collector and corresponding author), for her care and constant support to him.

## Supplementary Material

XML Treatment for Micropeplus liweiae
